# Social thermoregulation as a potential mechanism linking sociality and fitness: Barbary macaques with more social partners form larger huddles

**DOI:** 10.1038/s41598-018-24373-4

**Published:** 2018-04-17

**Authors:** Liz A. D. Campbell, Patrick J. Tkaczynski, Julia Lehmann, Mohamed Mouna, Bonaventura Majolo

**Affiliations:** 1 Moroccan Primate Conservation Foundation, Azrou, Morocco; 2 School of Psychology, University of Lincoln, Lincoln, United Kingdom; 3 CRESIDA, University of Roehampton, London, United Kingdom; 4 Max Plank Institute for Evolutionary Anthropology, Leipzig, Germany; 5 Institut Scientifique, Mohammed V University, Rabat, Morocco

## Abstract

Individuals with more or stronger social bonds experience enhanced survival and reproduction in various species, though the mechanisms mediating these effects are unclear. Social thermoregulation is a common behaviour across many species which reduces cold stress exposure, body heat loss, and homeostatic energy costs, allowing greater energetic investment in growth, reproduction, and survival, with larger aggregations providing greater benefits. If more social individuals form larger thermoregulation aggregations due to having more potential partners, this would provide a direct link between sociality and fitness. We conducted the first test of this hypothesis by studying social relationships and winter sleeping huddles in wild Barbary macaques (*Macaca sylvanus*), wherein individuals with more social partners experience greater probability of winter survival. Precipitation and low temperature increased huddle sizes, supporting previous research that huddle size influences thermoregulation and energetics. Huddling relationships were predicted by social (grooming) relationships. Individuals with more social partners therefore formed larger huddles, suggesting reduced energy expenditure and exposure to environmental stressors than less social individuals, potentially explaining how sociality affects survival in this population. This is the first evidence that social thermoregulation may be a direct proximate mechanism by which increased sociality enhances fitness, which may be widely applicable across taxa.

## Introduction

Many species exhibit highly differentiated social relationships between individuals (e.g. mammals^1^, birds^2^). Investment in social relationships detracts from time that could be spent on other activities, such as foraging, suggesting there is adaptive value to investing in social relationships. In a variety of species, research is emerging that individuals with more or stronger social relationships experience fitness benefits such as increased longevity^3,4^, increased likelihood of surviving extreme events^5–7^, increased birth rates^8,9^, and increased offspring survival and longevity^8–11^. The proximate mechanisms by which social relationships enhance fitness, however, are not entirely clear. Research into this topic has thus far focused on attenuation of physiological stress^12,13^, increased tolerance in intraspecific competition^14–16^, provision of social support in agonistic interactions^17–19^, protection from intraspecific harassment and infanticide^8,20^ and increased cooperation against predators^21^. However, a potential mechanism linking sociality and fitness that has thus far been overlooked but could apply to a wide range of species is social thermoregulation. Social thermoregulation is the cooperative promotion of body heat conservation, employed by a wide variety of species, including reptiles, birds and mammals, to cope with cold stress^22^. Social thermoregulation, including huddling, communal nesting and communal roosting, reduces exposure to environmental stressors and loss of body heat, thereby reducing body temperature fluctuations and physiological stress^22^. For endothermic animals, social thermoregulation provides substantial energetic savings due to reduced need for metabolic heat production, allowing more energy to be allocated to growth, development, competition, reproduction and parental care, and increasing the likelihood of surviving periods of energetic deficit^22^. To reduce potential costs of stress and aggression from close proximity, individuals may form thermoregulation aggregations primarily with closely bonded social partners^23,24^. If so, more social individuals may have more potential thermoregulation partners and alternative options if a particular partner is unavailable, allowing larger, thus more effective^22^, social thermoregulation aggregations. If so, this would provide a direct mechanistic link between individual sociality and fitness. Supporting this, research on core body temperatures of vervet monkeys (*Chlorocebus pygerythrus*) found that individuals with more social partners experience thermal benefits, including higher minimum and average body temperature and reduced heterothermy^25,26^, though these studies did not collect behavioural data on social thermoregulation so whether this is the mechanism responsible remains unclear.

In this study, we conduct the first test of the hypothesis that social thermoregulation is a potential proximate mechanism by which individual sociality affects fitness by investigating whether more social Barbary macaques (*Macaca sylvanus*) participate in larger social thermoregulation sleeping huddles. Barbary macaques inhabit mountainous environments with cold, snowy winters, which place them under considerable energetic stress^27,28^. Barbary macaques sleep in trees, when available, often forming sleeping clusters of multiple huddling individuals^29,30^. Previous research in this population found that sociality confers profound fitness benefits: during an extreme winter with substantial mortality, macaques with more social partners were more likely to survive, though the mechanism responsible was unknown^5,6^. We elaborate upon this research by testing whether social thermoregulation could explain the greater survival experienced by more social Barbary macaques^5,6^, and greater fitness experienced by more social individuals more generally^3,4,7–11^.

To test our hypothesis, we first validated two central assumptions. Firstly, research in both field and laboratory conditions has shown in a variety of taxa that larger social thermoregulation aggregations more effectively reduce body heat loss and thus provide greater thermoregulatory and energetic benefits^22^. We validate this in our study by investigating the effect of weather conditions on macaque huddle sizes, with the prediction that larger huddles will form under conditions that impose greater thermoregulatory demands. Secondly, we validate whether social relationships predict Barbary macaque huddling relationships, as in other species^23,24^. Finally, we test our main hypothesis that more social individuals (i.e. individuals with more or stronger social relationships) participate in larger huddles. If so, this would provide a potential proximate mechanism by which increased investment in social relationships confers a fitness advantage, which may be widely applicable across the large range of species that display differentiated social relationships and employ social thermoregulation.

## Results

Two groups of wild Barbary macaques (Blue Group and Green Group) were studied during winter from January to April 2015. For consistency with previous research on the fitness benefits of sociality in this population^5,6^, we focused only on social, and thus huddling, relationships between adults; juveniles were not included as study subjects.

### Assumption 1: Huddle sizes respond to thermoregulatory challenges

**Figure 1:**
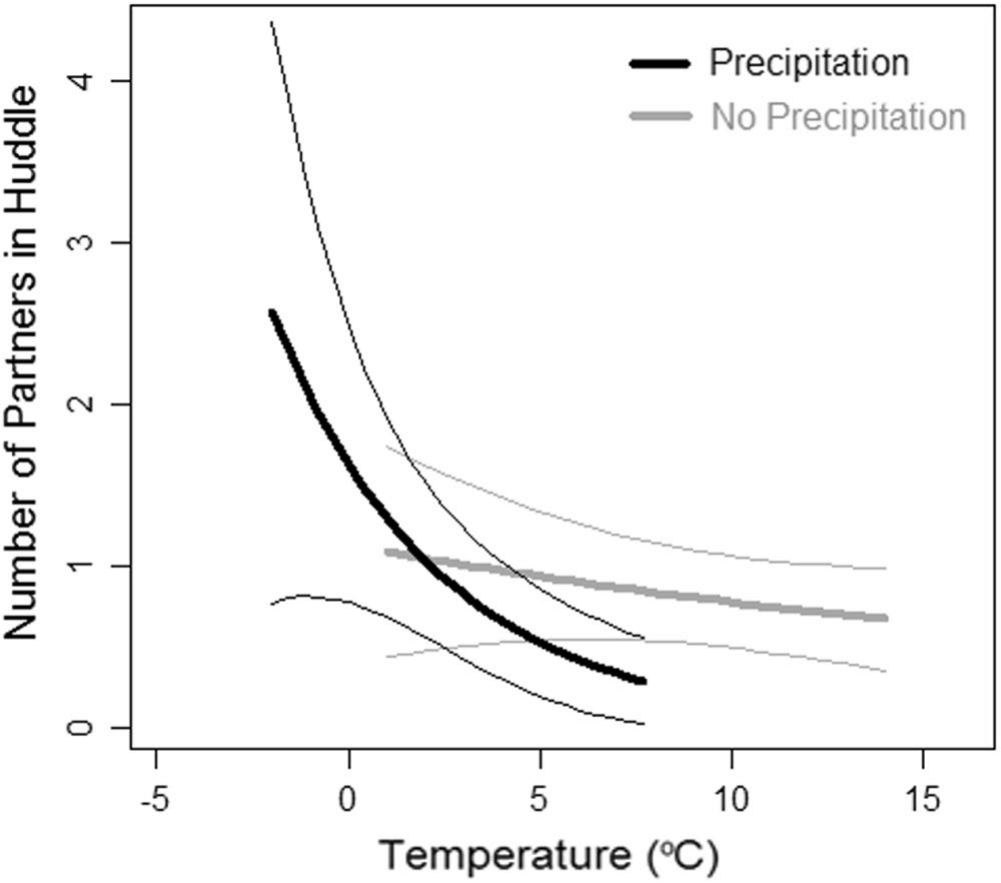
Predicted effect (thick lines) ± 95% CI (thin lines) from GLMM of the effect of weather (temperature and precipitation) on huddle size (the number of partners in a huddle).

As an indirect measure of whether huddle size affects thermoregulation, we tested whether huddle size increases under weather conditions that impose greater demands on metabolic heat production. The number of adults in a sleeping huddle ranged from 1 to 4 (mean = 1.6, SD = 0.7, N = 150). To avoid zero-truncation, the number of partners in a huddle (huddle size - 1) was used as the response variable. The number of partners in a huddle therefore ranged from 0 (an individual did not huddle with another subject on a given night) to 3 (an individual huddled with three others; mean = 0.6, SD = 0.7, N = 150). A GLMM controlling for date as a random effect found that the number of huddle partners was significantly affected by the interaction between temperature and precipitation (Table 1): larger huddles formed under the combined effect of low temperature and precipitation (Fig. 1). There was a significant difference between groups in the number of partners huddled with, likely due to differences in group composition: the number of adults in a huddle was lower in the group with few adults relative to juveniles.

**Table 1:** Results from GLMM investigating the relationship between huddle size (the number of partners in a huddle) and weather, controlling for study group.

Parameter	Estimate (SE)	*T*	*P*
Intercept	−0.08 (0.31)	−0.27	0.789
Group	−0.40 (0.19)	−2.11	0.036*
Temperature	−0.04 (0.03)	−1.19	0.238
Precipitation	0.37 (0.37)	0.99	0.323
Interaction Temperature: Precipitation	−0.19 (0.08)	−2.42	0.017**

Blue group was used as the baseline level for group. N = 150 huddles.

### Assumption 2: Social relationships predict huddle relationships

Multiple Regression Quadratic Assignment Procedure (MRQAP), a multiple regression procedure performed on dyads within multiple matrices^31^, found that in both groups, grooming relationships (the proportion of time from focal observations that individuals in a dyad groomed one another) predicted huddle relationships (the proportion of nights a dyad huddled together), while sex similarity and dominance rank difference did not significantly affect huddle relationships (Table 2).

**Table 2:** Results from MQRAP models predicting sleeping huddle relationships from grooming relationships, sex similarity, and dominance rank difference.

Parameter	Estimate	*P*
***Blue Group***		
Intercept	0.14	0.286
Grooming Relationship	0.88	<0.001 ***
Sex Similarity	−0.09	0.532
Dominance Rank Difference	−0.09	0.302
***Green Group***		
Intercept	0.01	0.977
Grooming Relationship	0.31	0.009 **
Sex Similarity	0.36	0.058
Dominance Rank Difference	−0.13	0.332

N = 132 dyads Blue Group, 210 dyads Green Group.

### Main Hypothesis: Individual sociality positively affects social thermoregulation huddle size

**Figure 2:**
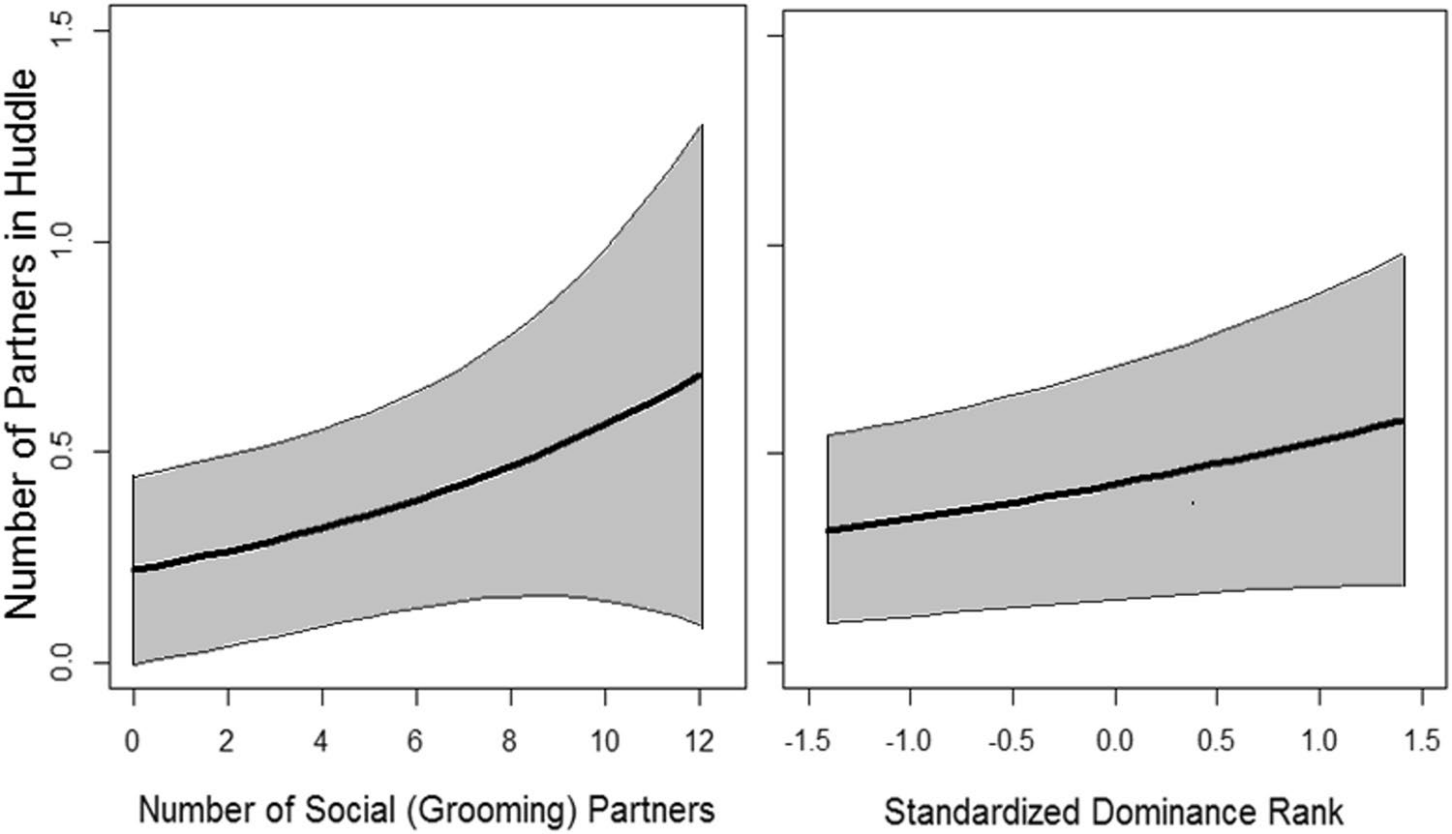
Predicted effect (thick lines) ± 95% CI (grey area) from GLMM of (**a**) the number of social (grooming) partners and (**b**) standardized dominance rank on huddle size (the number of partners with which an individual huddled). More dominant ranks are represented by larger values.

Subjects had a range of 1 to 12 social partners (the number of adults each subject groomed with), with an average of 7.0 ± 2.8 (SD). A GLMM found that the number of partners a subject huddled with on a particular night (N = 194 observations) was significantly affected by the number of social partners the subject had: monkeys with more social partners formed larger huddles (Fig. 2a). The collective strength of an individual’s social relationships (the total proportion of observation time each subject spent grooming with all social partners) did not affect huddle size (Table 3). Dominance rank also had a positive effect, with dominant individuals forming larger huddles than subordinates (Fig. 2b). These results were found while controlling for the significant effects of study group and weather (precipitation and low temperature) identified in the previous analysis (Assumption 1) and controlling for subject ID and date as random effects.

**Table 3:** Results from GLMM of variables affecting the number of individuals with which a subject huddled.

Parameter	Estimate (SE)	*T*	*P*
Intercept	−0.25 (0.36)	−0.67	0.501
Number of Social (Grooming) Partners	0.09 (0.04)	2.38	0.018*
Collective Strength of Social (Grooming) Relationships	−0.57 (0.46)	−1.23	0.221
Standardized Dominance Rank	0.22 (0.07)	3.33	0.001**
Sex	0.06 (0.18)	0.34	0.736
Group	−0.45 (0.18)	−2.50	0.013*
Temperature	−0.04 (0.02)	−2.02	0.045*
Precipitation	0.31 (0.30)	1.03	0.305
Interaction Temperature: Precipitation	−0.18 (0.08)	−2.32	0.022*

Larger values for dominance rank represent a more dominant individual. Female was used as the baseline level for sex. Blue Group was used as the baseline level for group. N = 194 observations.

## Discussion

Our research provides the first evidence that social thermoregulation is a potential proximate mechanism by which sociality affects fitness. While an increasing number of studies are finding that more social individuals experience a fitness advantage^3–11^, our study is the first to suggest that social thermoregulation could be a mechanism explaining these effects. The prevalence of social thermoregulation across taxa presents the possibility that this is a widespread mechanism by which individual sociality affects fitness.

The thermoregulatory and energetic benefits provided by huddling increase with the number of individuals involved because more partners better reduce exposed surface area and thus heat loss^22^. Although we did not measure thermoregulation directly, the effect of temperature and precipitation on huddle sizes in our study supports that this is also the case for wild Barbary macaques. Huddle sizes increased with low temperature and precipitation, which would allow macaques to counteract high thermoregulatory costs by more effectively conserving heat and thus energy^22^. This agrees with hormonal data in this population suggesting that rain and low temperatures increase the metabolic rate of macaques^28^.

Barbary macaques huddled with their social (i.e. grooming) partners, similar to other macaque species^23,24,32^. However, unlike other macaque species (e.g. *M. radiata*^33^, *M. fuscata*^23,34^, *M. thibetana*^24^), rank difference and sex similarity did not significantly influence huddling relationships; Barbary macaques were no more or less likely to huddle with individuals of similar sex or rank, independently of their social relationship. The number of potential huddle partners an individual has, therefore, appears to be dependent on the number of social relationships actively formed through grooming, rather than their sex or rank.

As huddle size influences thermoregulation and energetics^22^, the finding that individuals with more social relationships formed larger huddles therefore suggests that more social individuals experience reduced exposure to cold stress and reduced energy expenditure on body temperature maintenance, which can directly benefit fitness^22^. Individuals that better conserve energy would be more likely to survive periods of winter energy stress^27,28^, providing a mechanism to explain why Barbary macaques with more social partners experienced greater survival during an extreme winter^5,6^. Reducing exposure to environmental stress can further increase fitness by reducing physiological stress, which can negatively impact health, immunity, reproduction and survival if chronically elevated^35–37^. Low temperatures increase glucocorticoid metabolite levels in Barbary macaques but less so in males with stronger social relationships^13^, which the results presented here suggest could be due to participation in more effective social thermoregulation. Our results therefore strongly support the hypothesis that social thermoregulation is a mechanism by which individual sociality affects fitness.

Despite the benefits of larger huddles, the maximum huddle size observed in this study and in previous studies on Barbary macaques^29^ were small. As a relatively large-bodied species with arboreal sleeping habits^38^, the size and mass of sleeping huddles is likely restricted by the size and strength of branches. This is supported by other research on sleeping area selection in these groups [Campbell *et al*., in prep]. The formation of strong relationships may therefore be particularly important if Barbary macaques must be selective in the choice of huddle partners on a particular night. Dyads with stronger grooming relationships were more likely to huddle together, but the collective strength of an individual’s social relationships did not influence huddle size. This is consistent with previous findings that the quantity but not quality of social relationships affects Barbary macaque winter survival^5^ and variations in core body temperature in vervet monkeys^25^. Thus, the availability of multiple alternative thermoregulation partners appears to be more valuable for social thermoregulation than overall strength of social relationships. Dominance rank also had a slight positive effect on huddle size, which could be due to high-ranking individuals displacing subordinates from higher-quality sleeping branches that allow larger huddle size and mass [Campbell *et al*., in prep]. However, rank did not affect individual survival during an extreme winter^5,6^ so rank alone may be unable to provide thermoregulation benefits if an individual does not also have a suitable network of huddle partners.

Though this study was conducted in an extreme climate, major cold stress may not be a requirement for this mechanism to give more social individuals a fitness advantage. Huddling, communal nesting and communal roosting are common behaviours employed by a wide variety of mammals, birds and reptiles across a range of habitats in response to lower night temperatures, poor weather conditions and during periods of reduced thermogenic capacity such as when molting^22^. Even a small advantage provided to more social individuals through participation in more effective social thermoregulation could conceivably provide fitness benefits over the long term when engaged in regularly, or even nightly, as social thermoregulation often is^22^. In the ecological conditions of this study where winter energy availability is severely limited, energetic benefits provided by social thermoregulation may explain survival benefits afforded by sociality^5,6^. In less extreme climates, energetic benefits afforded by more effective social thermoregulation could contribute to the greater longevity, reproductive output, and offspring survival observed in other studies^3,4,7–11^ by allowing greater energetic investment in growth and reproduction^22^.

This study provides the first evidence that individual sociality affects social thermoregulation. Individuals that invest in more social relationships can participate in larger and thus more effective social thermoregulation aggregations due to having more potential partners, thereby suggesting a direct thermoregulatory, energetic, and physiological mechanism by which increased sociality enhances fitness. Similar mechanisms may be applicable across a wide range of species that employ social thermoregulation^22^ and display non-random social associations^1,2^. This adds to our understanding of the benefit of forming and maintaining social bonds and thus the evolution of complex animal sociality.

## Methods

Research was purely observational and performed in accordance with all relevant guidelines and regulations. Research was approved by the Kingdom of Morocco *Haut Commissaire aux Eaux et Forêts et à la Lutte Contre la Désertification* (Permit N°44/2014) and the University of Roehampton Ethics Committee (LSC 13/088).

### Study Site

This study was conducted in Ifrane National Park, Middle Atlas Mountains, Morocco (33° 24′N, 05° 12′W, average altitude 1800 m), from January 6 to April 17 2015. The forest of the study site is composed of predominantly Atlas cedar (*Cedrus atlantica*) and holm oak (*Quercus ilex*). Snow cover was continuous during the study period from January until the end of March, then intermittent throughout the remainder of the study period. Winter conditions of the area have been described in previous publications^5,27^.

### Subjects

Both study groups were fully habituated to observation and all adults could be individually recognized by physical features. Blue Group consisted of 25 individuals and contained few juveniles relative to adults (five adult males (6+ years of age), seven adult females (5+ years of age), nine juveniles and four infants (<1 years of age)). Green Group consisted of 42 individuals and contained many juveniles relative to adults (seven adult males, eight adult females, twenty-one juveniles and six infants).

### Data Collection

Social data were collected for a total of 98 days (47 for Blue Group, 51 for Green Group). Thirty-minute continuous focal observations were conducted on all adults for a total of 471 hours (208.5 hours for Blue Group, 262.5 hours for Green Group), recording the identities of all adult social partners, duration of time allo-grooming, and the frequency and direction of all aggression (threats, non-contact aggression and contact aggression) and dominance interactions (e.g. presenting submission in the absence of aggression; Supplementary Materials Table S1). Dyadic aggression and dominance interactions between adults with a clear outcome (one individual was aggressive and the other was submissive) were also recorded *ad libitum* to measure dominance hierarchies. Groups were followed from approximately 6:00 to 19:00 each day, with focal sampling performed from approximately 8:00 to 18:00. All subjects were observed for three sessions per week, with focal order selected by randomization without replacement. Mean observation time per subject during the study was 17.44 ± 0.31 (SD) hours of focal data (Blue Group 17.38 ± 0.23, Green Group 17.50 ± 0.38).

The groups were followed to sleeping sites in the evening and observers returned in the morning before sunrise to identify as many sleeping huddles as possible before macaques awoke and moved from sleeping positions. Studies in semi-free-ranging Barbary macaques found they rarely move from sleeping locations during the night^29,30^ and, when they do, they typically return to their original sleeping location and huddle arrangement^29^. Thus, the sleeping huddles in which monkeys were found in the morning are assumed to be where they remained throughout the night. Air temperature was recorded with handheld weather meters (factory calibrated Kestrel 3500 meter held one meter above ground in shade in the centre of the sleeping site) in the evening when macaques entered their sleeping trees (an average of 41 ± 17 minutes before sunset) and in the morning when they exited their sleeping trees (an average of 28 ± 15 minutes after sunrise) and it was recorded whether there was precipitation overnight at the location of the sleeping site at any time between when macaques entered and exited their sleeping trees (yes/no). Data on sleeping huddle composition were collected over a total of 52 nights (27 nights for Blue Group, 25 nights for Green Group), recording a total of 267 observations of adult sleeping arrangements (144 Blue Group, 123 Green Group). Each adult dyad in the Blue Group was observed an average of 18.2 ± 2.5 nights and each dyad in the Green Group was observed an average of 13.3 ± 4.1 nights.

Data collection only began when all observers (PJT, LADC, and 3 assistants) achieved significant inter-observer reliability in subject identification and frequency and durations of recorded behaviours, as judged by two consecutive 30-minute inter-observer reliability test focals with PJT with significant intra-class correlation coefficients >0.95 (*P* < 0.05)^39^.

### Analysis

Analyses were performed in R version 3.2.3^40^.

### Dominance Hierarchies

Dominance hierarchies were calculated to assess the potential effects of dominance rank on social thermoregulation. All dyadic interactions of aggression and dominance interactions from focal and *ad libitum* sampling with a clear outcome were used to calculate dominance hierarchies (Blue Group N = 447, Green Group N = 369) using an Elo-rating procedure^41^ with the R package EloRating^42^. Calculations of hierarchy stability (S), which ranges from 1 (completely stable) to 0 (completely unstable), found all dominance hierarchies to be stable during the study period (Blue Group males S = 0.98, females S = 0.99, Green Group males S = 0.98, females S = 0.99). Dominance ranks were standardized for comparison across sexes and across groups and multiplied by -1 such that a larger value represents a more dominant rank.

### Social networks

Social networks were created to quantify social relationships. Undirected interaction matrices were created for grooming and huddling relationships using the simple ratio index^43^. Grooming relationships were calculated as the total duration of time a dyad spent grooming together divided by the total observation time of both individuals (observation time individual A plus observation time individual B). Huddle relationships were defined as the number of nights a dyad huddled together divided by the number of nights either monkey in the dyad was observed. Relationship values therefore ranged between 0 (dyad never groomed/huddled) and 1 (dyad always groomed/huddled). The R package igraph^44^ was used to create social networks and extract for each subject the number of grooming partners (*degree* measure in igraph) and collective strength of grooming relationships (*strength* measure in igraph). The number of grooming partners is the number of adults that a subject had been observed to groom with over the entire study period (the binary degree in the grooming social network^45^) and collective strength of grooming relationships is the sum of a subject’s grooming relationships across all adult social partners, i.e. the total proportion of time each subject spent grooming with their social partners (the node strength or weighted degree of the grooming social network^45^).

### Assumption 1: Huddle sizes respond to thermoregulatory challenges

The average of temperature measurements collected in the evening and morning was used in the analyses. Dates for which weather data were not available for both the evening and the morning (e.g. the group was not followed the previous evening) were not included in the analysis, resulting in 36 observation nights with weather data (22 nights Blue Group, 14 nights Green Group). Subjects were recorded in a total of 150 huddles on these nights (85 in Blue Group, 65 in Green Group).

A GLMM tested whether the number of partners in a huddle was affected by temperature, precipitation, or their interaction, while controlling for group as a fixed effect and date as a random effect. Due to low variation in the response variable (range = 0–3, mean = 0.6, SD = 0.7; Supplementary Material Fig. S1), the data were underdispersed according to a Poisson model (dispersion parameter = 0.71). A quasi-Poisson model was therefore fitted, which adjusts the standard error of the estimates according to the dispersion parameter of the Poisson model^46^. Analysis was done with the lme4 package in R^47^ following a full model approach^46^. Collinearity between explanatory variables was first assessed using pairplots and variance inflation factors (VIF) using R source code from Zuur *et al*.^46^, which found no strong collinearity (VIF < 2.0). Model validation followed protocols of Zuur *et al*.^46^ (Supplementary Material Fig. S2).

### Assumption 2: Social relationships predict huddle relationships

MRQAP models assessed for each group whether grooming relationships predict huddle relationships, while controlling for dominance rank (difference in rank between the two individuals of each dyad) and sex (binary similarity measure for each dyad: same-sex vs. different-sex). Because MRQAP is performed on matrices and individuals from different groups do not interact, the two study groups were analysed separately. Matrices were standardized (mean subtracted and divided by the standard deviation) to allow comparison between the two groups of different sizes. Collinearity between explanatory matrices (dyadic grooming relationship values, dominance rank difference and sex similarity) was first assessed with Mantel tests using the R package vegan^48^, which found no strong collinearity between the explanatory matrices. MRQAP analysis was performed with the asnipe package^49^ in R using 10,000 permutations.

### Main Hypothesis: Individual sociality positively affects social thermoregulation huddle size

A GLMM tested whether the number of partners a subject huddled with on a particular night was affected by the number or strength of its social relationships, while controlling for weather, dominance rank and group as fixed effects and subject ID and date as random effects. Due to low variation in the response variable (range = 0–3, mean = 0.8, SD = 0.7, N = 194, Supplementary Material Fig. S3), the data were underdispersed according to a Poisson model (dispersion parameter = 0.49) and therefore a quasi-Poisson model was used^46^. There was no strong collinearity between explanatory variables (VIF < 2.4^46^). Analysis was done with the lme4 R package^47^. Model validation^46^ confirmed that the model fit the data well (Supplementary Material Fig. S4). To ensure results of this analysis were reliable despite the underdispersion, the supplementary materials include a binary analysis (whether or not the subject huddled with at least one adult huddle partner), which avoids underdispersion (Supplementary Material Table S1, Fig. S5). The results of the binary analysis confirmed the results of the count analysis: the number of social partners significantly affected social thermoregulation while the collective strength of social relationships did not. Individuals with fewer social partners were more likely to sleep without a huddle partner than individuals with more social partners.

### Data availability

The data used in this study are available at: http://eprints.lincoln.ac.uk/30497/.

## Electronic supplementary material

Supplementary Materials
