# Supplementary material for: Social thermoregulation as a potential mechanism linking sociality and fitness: Barbary macaques with more social partners form larger huddles
Source: Sci Rep. 2018 Apr 17;8:6074. doi: 10.1038/s41598-018-24373-4 (PMC5904170; doi:10.1038/s41598-018-24373-4)
Supplement: Supplementary file 1 — Supplementary Materials [file 41598_2018_24373_MOESM1_ESM.pdf]

Supplementary Materials

Social thermoregulation as a potential mechanism linking sociality and fitness: Barbary macaques with more social partners form larger huddles

Liz AD Campbell[1,2], Patrick J Tkaczynski[3,4], Julia Lehmann[3], Mohamed Mouna[5], Bonaventura Majolo[2]

[1] Moroccan Primate Conservation Foundation, Azrou, MA
[2] School of Psychology, University of Lincoln, Lincoln, UK
[3] CRESIDA, University of Roehampton, London, UK
[4] Max Plank Institute for Evolutionary Anthropology, Leipzig, DE
[5] Institut Scientifique, Mohammed V University, Rabat, MA

Corresponding author: Liz AD Campbell, lizadcampbell@gmail.com

Definitions of aggression and dominance behaviours used to determine dominance rank hierarchies are provided in Table S1. The distribution of the number of adults that each monkey huddled with was underdispersed according to a Poisson model for both analyses of huddle size, Assumption 1 (Fig. S1) and the Main Hypothesis (Fig. S3), so quasi-Poisson models were used. The same dataset was used for these two analyses, but Assumption 1 was analysed by huddle (N = 150 huddles) to avoid pseudo-replication of huddles that contained more than one subject, while the Main Hypothesis was analysed by subject (N = 194 observations of subjects) to assess the effects of individual sociality. In 17 huddles included in Assumption 1 the subjects were counted but not identified with full confidence and thus were not included in the other analyses. The random effects of the Poisson GLMM models had 0 variance, indicating there was no residual correlation according to date and subject that was not accounted for by the fixed effects, allowing use of the quasipoisson adjustment in the lme4 package in R[1]. To ensure the results of the Main Hypothesis were robust despite underdispersion in the response variable, data were also analysed as a binary response variable (i.e. whether the subject huddled with at least one adult huddle partner) to avoid underdispersion. Because larger huddles, including both adults and juveniles, are more likely to contain at least one adult huddle partner, total huddle size including both adults and juveniles was included as a control variable in the binary analysis. The results of the binary analysis shown in Table S2 confirm the results of the count analysis in the main manuscript: the number of social partners positively affected social thermoregulation while the collective strength of social relationships did not. Individuals with few social partners were more likely to sleep without a huddle partner than individuals with more social partners.

Model validation followed the procedure described by Zuur *et al.*[2] Graphs of model fit, showing raw data and data simulated from the models, are shown in Fig. S2 (Assumption 1), S4 (Main Hypothesis count analysis) and Fig. S5 (Main Hypothesis binary analysis).

Table S1: Definitions of aggression and dominance behaviours used to measure dominance rank hierarchies. All bouts of dyadic aggression and dominance interactions between adults were recorded, including the identities of both individuals involved and the direction.

Behaviour	Definition
**Threats**	
Open Mouth	A moderate threat display in which the head is lowered and stuck forward towards the individual at which the display is intended, the eyebrows are lifted and the mouth is opened into an ‘O’ shape with the lips covering the teeth. It may be accompanied by a “hoo” panting sound.
**Non-Contact Aggression**	
Lunge	Monkey makes a sudden intense movement towards another monkey but does not move a large distance
Charge	Monkey charges at another monkey for less than 5 metres
Chase	Monkey chases another at high speed for a long distance (> 5 m)
**Contact Aggression**	
Grab	Monkey grabs the body of another
Slap	Monkey hits another monkey with an opened hand
Bite	Monkey bites another
Jump on	Monkey jumps onto another
Push	Monkey pushes another with its hands or feet
Push & Pull	Monkey grabs hold of another monkey and makes a shaking movement
**Dominance Interactions**	
Supplant	A dominant individual approaches a subordinate and the subordinate moves away to create distance between them. The dominant individual may or may not then take the physical space that had been occupied by the subordinate.
Present Submission	Monkey presents its hindquarters to another in submission.
Bared teeth grimace	A submissive or nervous display where the mouth is open and lips pulled up so that the teeth and gums are showing, the eyebrows are raised and ears flattened against the head.

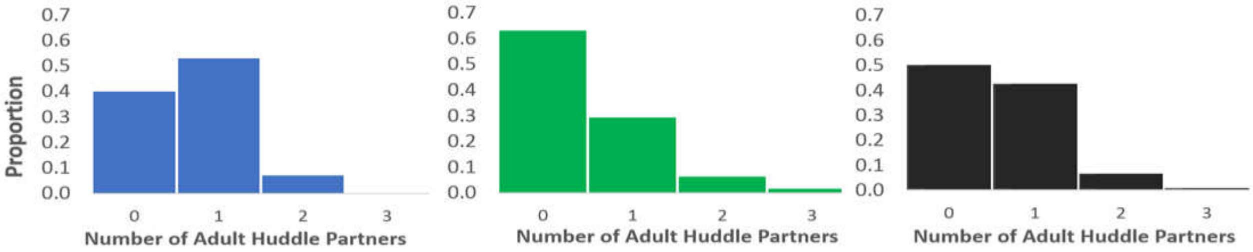

Figure S1: Count distribution of the response variable for Assumption 1 (analysed by huddle), showing the number of huddle partners (huddle size - 1) as a proportion of the total number of huddles in (a) Blue Group (N = 85), (b) Green Group (N = 65), and (c) in total (N = 150).

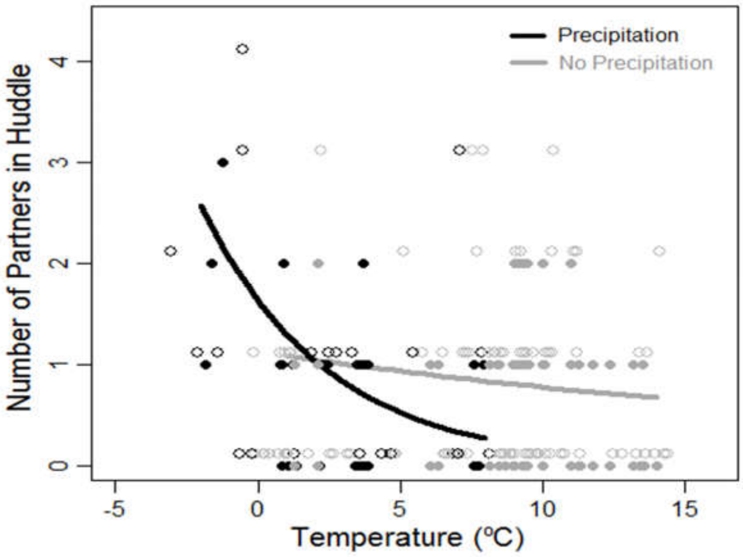

Figure S2: Graph of model fit for Assumption 1, showing raw data (solid points), simulated fitted values (open circles) and fitted lines for the effects of weather on huddle size (number of partners in huddle). Random variation has been added to data points along the x-axis and simulated values are shown slightly above the raw data to aid visualization of points with the same value.

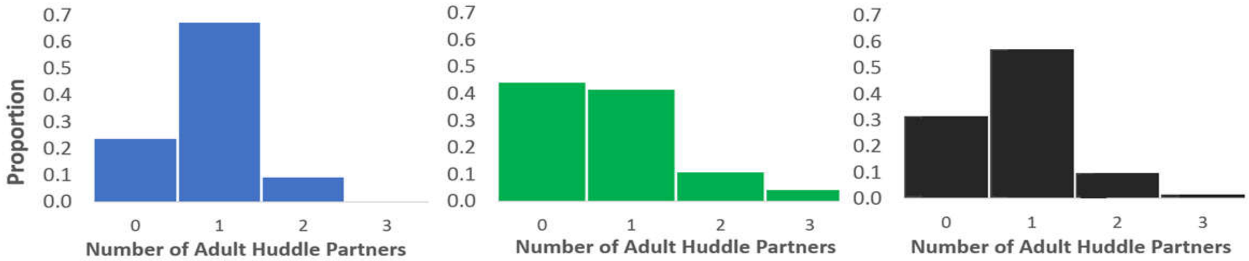

Figure S3: Count distribution of the response variable for the Main Hypothesis (analysed by subject), showing the number of partners each focal subject huddled with as a proportion of total observations in (a) Blue Group (N = 119), (b) Green Group (N = 75), and (c) in total (N = 194).

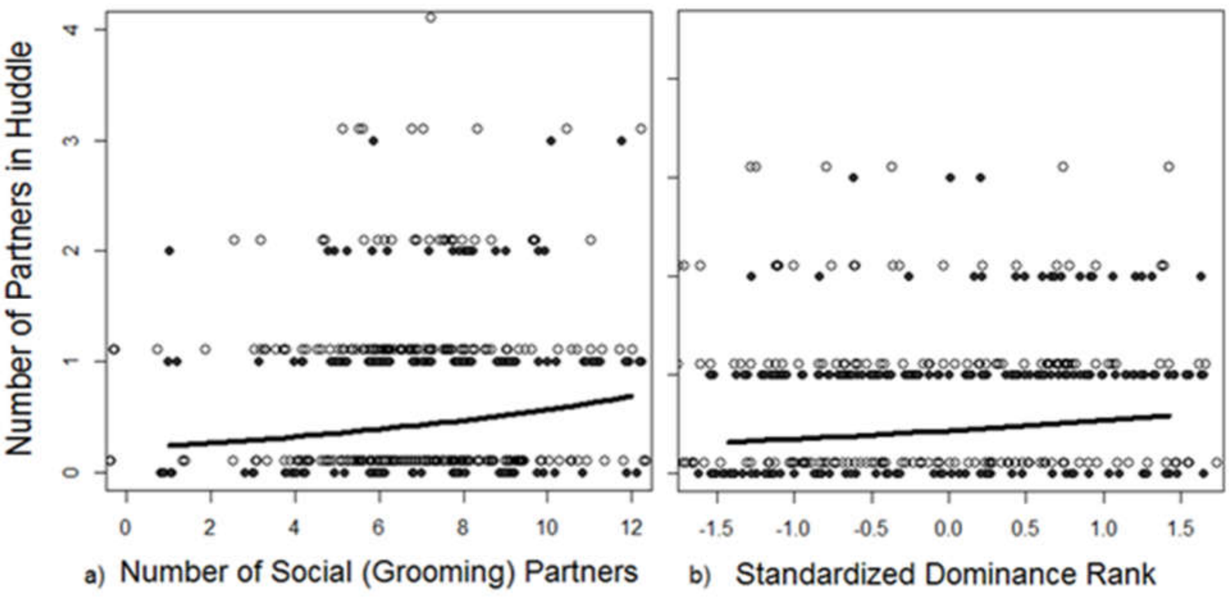

Figure S4: Graphs of count model fit for the Main Hypothesis, showing raw data (solid points), simulated fitted values (open circles) and fitted lines for the effects of a) the number of social partners and b) dominance rank on huddle size (the number of partners in huddle). Random variation has been added to data points along the x-axis and simulated values are shown slightly above the raw data to aid visualization of points with the same value.

Table S2: Results of logistic GLMM of the probability of a subject huddling with at least one other adult. Female was used as the baseline level for sex and Blue Group was used as the baseline level for group. N = 194 observation.

**Parameter**	**Estimate (SE)**	***Z***	***P***
Intercept	-1.85 (1. 94)	-1.24	0.215
Number of Social (Grooming) Partners	0.33 (0.14)	2.40	0.016 *
Strength of Social (Grooming) Relationships	-0.85 (2.01)	-0.42	0.672
Total Huddle Size (including juveniles)	2.09 (0.39)	5.35	< 0.001 ***
Dominance Rank	-0.08 (0.25)	-0.33	0.742
Sex	0.54 (0.65)	0.83	0.407
Group	-2.44 (0.72)	-3.40	< 0.001 ***
Temperature	-0.12 (0.09)	-1.31	0.192
Precipitation	-0.02 (1.35)	-0.01	0.991
Interaction Temperature : Precipitation	-0.12 (0.23)	-0.51	0.611

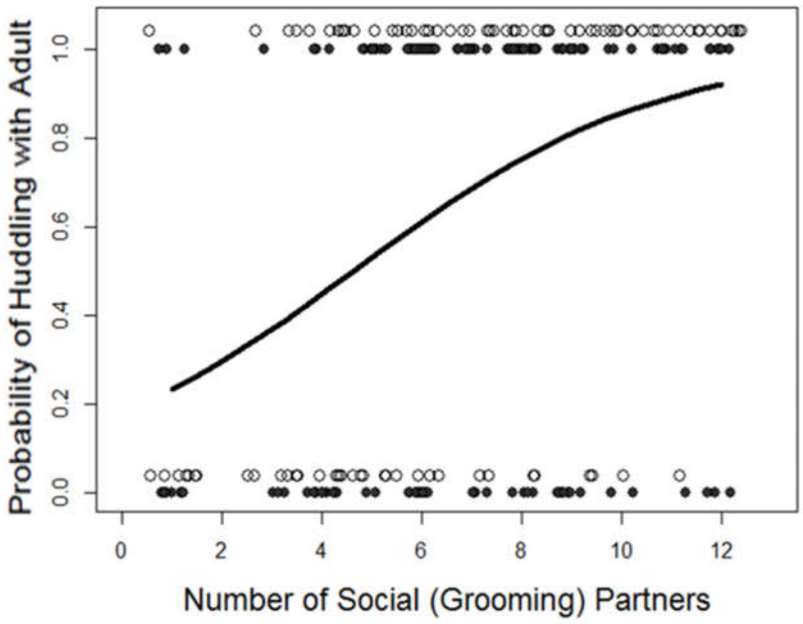

Figure S5: Graph of logistic model fit, showing raw data (solid points), simulated fitted values (open circles), and fitted line for the effects of the number of social (grooming) partners on the probability of huddling with at least one other adult. Random variation has been added to data points along the x-axis and simulated values are shown slightly above the raw data to aid visualization.

References

1. Bates D, Maechler M, Bolker B, Walker S. lme4: Linear mixed-effects models using Eigen and S4. R package version 1.1-7. http://CRAN.R-project.org/package=lme4 (2014).
2. Zuur AF, Hilbe JM, Ieno EN. *A Beginner's Guide to GLM and GLMM with R: A Frequentist and Bayesian Perspective for Ecologists*. Newburgh: Highland Statistics (2013).
